# Cell-free supernatant of *Levilactobacillus brevis* (RAMULAB51) from coconut inflorescence sap (Neera) enhances glucose uptake and PPAR-γ in 3T3-L1 adipocytes and inhibits α-glucosidase and α-amylase

**DOI:** 10.3389/fmicb.2024.1497023

**Published:** 2024-12-23

**Authors:** Chandana Kumari V B, Ramith Ramu, Prithvi S. Shirahatti, Perwez Alam, Ling Shing Wong

**Affiliations:** ^1^Department of Biotechnology and Bioinformatics, JSS Academy of Higher Education and Research, Mysore, Karnataka, India; ^2^St. Joseph’s College for Women, Rammanahalli, Karnataka, India; ^3^Department of Pharmacognosy, College of Pharmacy, King Saud University, Riyadh, Saudi Arabia; ^4^Faculty of Health and Life Sciences, INTI International University, Nilai, Malaysia

**Keywords:** Neera, probiotics, Type 2 Diabetes Mellitus, α-glucosidase, α-amylase, PPAR-γ activation, 3T3-L1 adipocytes

## Abstract

**Introduction:**

Lactic acid bacteria are prized for their probiotic benefits and gut health improvements. This study assessed five LAB isolates from Neera, with RAMULAB51 (*Levilactobacillus brevis*, GenBank ON171686.1) standing out for its high hydrophobicity, auto-aggregation, antimicrobial activity, and enzyme inhibition. It evaluated RAMULAB51’s potential in probiotics and diabetes management, focusing on its effects on digestive enzyme inhibition, glucose uptake, and adipocyte function.

**Methods:**

Isolates were characterized by Gram staining, catalase reaction, growth at 37°C, and tolerance to phenol, pH, and gastrointestinal conditions. Molecular identification using 16S rRNA sequencing. Evaluations included hydrophobicity, auto-aggregation, HT-29 cell line adhesion, antimicrobial activity, and antibiotic susceptibility. Enzyme inhibition was measured for α-glucosidase and α-amylase using cell-free supernatant, cell extract, and intact cells. Adipogenesis was assessed through Oil-Red O staining, gene expression analysis (PPAR-γ, C/EBPα, Adiponectin, Glut-4, FAS), and glucose uptake assays on 3T3-L1 cells.

**Results:**

All isolates showed good tolerance to pH (≤9.15 CFU/ml), phenol (≤9.90 CFU/ml), hydrophobicity (≤78.14%), and auto-aggregation (≤92.23%). RAMULAB51 demonstrated the highest tolerance, hydrophobicity, and auto-aggregation. It strongly co-aggregated with *Micrococcus luteus* and *Bacillus subtilis*, showing significant antimicrobial activity with a 24 mm inhibition zone against *Micrococcus luteus*. All isolates were sensitive to Ampicillin, Azithromycin, Streptomycin, and Tetracycline, but resistant to Methicillin and Vancomycin. RAMULAB51 demonstrated the highest enzyme inhibition: α-glucosidase (68.45% CFS, 60.18% CE, 42.15% IC) and α-amylase (80.74% CFS, 61.23% CE, 35.12% IC). By inhibiting these digestive enzymes, RAMULAB51 reduces the conversion of carbohydrates into glucose, thereby decreasing blood glucose levels. This reduction in circulating glucose subsequently influences adipocyte function, as evidenced by the enhanced glucose uptake (1000 µg/mL) and upregulation of PPAR-γ, C/EBPα, Adiponectin, and Glut-4, alongside the downregulation of FAS.

**Conclusion:**

The study highlights RAMULAB51’s potential for improving glucose and lipid metabolism. Further, *in vivo* research is needed to explore its full therapeutic benefits. These findings confirm RAMULAB51’s significant probiotic potential and its promise for diabetes management, warranting further clinical investigation.

## 1 Introduction

Coconut sap, commonly known as Neera, is a naturally sweet liquid harvested from the unopened flower buds of the coconut tree (*Cocos nucifera L.*) (Jose et al., 2018). Esteemed in South India as the “Tree of Heaven,” the coconut tree is renowned for its numerous applications, with Neera emerging as a particularly valued product for both its traditional and nutritional significance (Misra, 2016). This sap is enjoyed fresh or processed into various value-added products, such as palm sugar, jaggery, fermented beverages (toddy), and vinegar (Asha et al., 2019; Rajath and Santhoshkumar, 2020). Despite Neera’s well-established role in traditional medicine and its recognized nutritional profile, its potential as a source of probiotics remains relatively unexplored.

Neera’s traditional use encompasses a range of health benefits, addressing conditions such as bronchial suffocation, anemia, tuberculosis, and piles (Srinivasu et al., 2021). This refreshing sap is celebrated for its rich content in vitamins (such as B-complex), minerals (like potassium and magnesium), antioxidants, and bioactive compounds that are not all commonly found together in other sources of probiotics like dairy or typical fermented foods detected (Misra, 2016). Neera, per 100 mL, has a nutritional composition including total solids (15,200–19,700 mg), pH (4.0–4.6), specific gravity (1.059–1.076), total sugar (14,400 mg), original reducing sugar (5,580 mg), total reducing sugar (9,850 mg), total ash (110–410 mg), iron (15 mg), phosphorus (7,590 mg), citric acid (500 mg), ascorbic acid (16–30 mg), proteins (230–320 mg), and no alcohol detected (Misra, 2016). However, the potential of Neera as a probiotic source field that has received limited attention exciting new prospects (Somashekaraiah et al., 2019). Probiotics, are defined as live microorganisms that, when ingested in sufficient quantities, offer health benefits. They are essential for promoting gut health and supporting immune system regulation (Kim et al., 2021). Given Neera’s natural microbial content, it may harbor probiotic strains that could positively influence gut flora and overall health (Chinnamma et al., 2019).

The significance of probiotics in managing metabolic disorders, particularly diabetes, is increasingly recognized. Type 2 Diabetes Mellitus (T2DM) presents a major global health issue, marked by insulin resistance and disrupted glucose metabolism (Wondmkun, 2020). Probiotics have gained attention as a potential complementary therapy for diabetes management because of their capacity to enhance gut health, regulate immune function, and affect metabolic processes (Han and Lin, 2014). Probiotics have been shown to enhance insulin sensitivity, reduce inflammation, and improve glycemic control, making them valuable in managing diabetes and related conditions. Neera’s natural microbial diversity provides a unique environment that might support the growth of specialized microbial communities. This environment may influence *Lactobacillus* spp. to develop adaptations, such as increased resistance to acidic and bile conditions in the human gut, which enhances probiotic effectiveness (Brunkwall and Orho-Melander, 2017; Tomusiak-Plebanek et al., 2018; Yang et al., 2020).

One mechanism through which probiotics exert their beneficial effects involves the modulation of enzyme activity and glucose metabolism. Key enzymes such as α-glucosidase and α-amylase are central to carbohydrate digestion (Shai et al., 2011). α-glucosidase, located in the small intestine, breaks down complex carbohydrates into glucose, while α-amylase initiates carbohydrate digestion in the mouth and stomach (Panwar et al., 2014). Inhibiting these enzymes can slow glucose absorption, thus managing postprandial blood sugar levels. Also, antioxidants in Neera might aid in reducing oxidative stress, a factor linked to diabetes progression. This combination may increase the health benefits of the *Lactobacillus* strain isolated from Neera. Probiotics with enzyme-inhibitory properties could therefore play a significant role in diabetes management by moderating glucose absorption and reducing blood sugar spikes (Huligere et al., 2023).

However, managing hyperglycemia through enzyme inhibition is only part of the strategy; understanding how probiotics influence adipocyte differentiation is also crucial for comprehensive diabetes management. Peroxisome proliferator-activated receptor gamma (PPAR-γ) is a crucial transcription factor involved in adipocyte differentiation and function. PPAR-γ regulates genes associated with adipogenesis, glucose metabolism, and insulin sensitivity (Chandra et al., 2008). Activation of PPAR-γ promotes the differentiation of preadipocytes into mature adipocytes and enhances fatty acid storage (Kim et al., 2020). It also plays a role in improving insulin sensitivity and maintaining glucose homeostasis (Shen et al., 2014). The analysis of how natural compounds or probiotics influence PPAR-γ activity could provide new strategies for the management of metabolic disorders such as diabetes (Kim et al., 2011). Modulating PPAR-γ activity could influence fat metabolism, glucose uptake, and insulin sensitivity, offering potential therapeutic benefits.

A valuable model for studying adipocyte differentiation and function is 3T3-L1 adipocytes, commonly used in research. These cells can be induced to differentiate into mature adipocytes, making them ideal for investigating processes such as glucose metabolism and the effects of various compounds on adipogenesis (Rizzatti et al., 2013). Evaluating glucose uptake in 3T3-L1 adipocytes is critical for understanding the impact of Neera’s probiotic components on glucose metabolism. Enhanced glucose uptake by adipocytes is a key indicator of improved insulin sensitivity and better glycemic control (Yagi et al., 2020). Assessing how Neera’s probiotic content influences glucose uptake can provide insights into its potential benefits for diabetes management.

The investigation of Neera for its probiotic potential represents a unique opportunity to expand its role beyond traditional uses and explore its impact on metabolic health. By examining Neera’s effects on α-glucosidase and α-amylase inhibition, PPAR-γ activation, and glucose uptake in 3T3-L1 adipocytes, this study aims to offer a comprehensive understanding of its potential benefits for diabetes management. The findings could pave the way for novel, natural approaches to managing diabetes and improving metabolic health. Moreover, this research may highlight the broader implications of incorporating Neera into modern diets, not only as a traditional beverage but also as a functional food with significant health benefits. Increased awareness and consumption of Neera could enhance its recognition as a valuable component in the fight against diabetes and related metabolic disorders.

## 2 Methodology

### 2.1 Experimental design

The experimental design for this study included isolating and characterizing lactic acid bacteria (LAB) from Neera samples obtained from *Cocos nucifera* under sterile conditions and transported at 4°C. LAB was isolated by plating diluted samples on MRS agar, then biochemically characterized using Gram staining and catalase activity tests. Molecular identification was accomplished using PCR amplification of the 16S rRNA gene. Bile salt and simulated gastrointestinal tolerance were used to measure probiotic qualities, as well as adhesion capacity by cell surface hydrophobicity and coaggregation experiments. Antibacterial activity was assessed using the agar well diffusion method, and antibiotic susceptibility was tested using the disc diffusion method. In addition, enzyme inhibition experiments for α-glucosidase and α-amylase were performed, and 3T3-L1 preadipocytes were cultivated to investigate the effects of LAB on preadipocytes were cultured to evaluate the effects of LAB on cell viability and differentiation. Statistical analysis was performed using one-way ANOVA followed by Duncan’s multiple range test, with significance set at *p* < 0.05, and all experiments were conducted in triplicate.

### 2.2 LAB strains biochemical assay and culture condition

LAB strains were isolated from natural Neera samples obtained from *Cocos nucifera* (coconut tree) using the method (Somashekaraiah et al., 2019). In this study, Neera was collected in the early spring of March at 4:30 AM to maintain its freshest state, minimizing microbial and biochemical changes from fermentation due to sunlight exposure, thereby preserving its native microbial profile and nutrient composition, which is essential for accurate LAB strain isolation and characterization. Additionally, Neera was sampled from a single coconut tree in Mandya, Karnataka, India, to ensure controlled conditions and minimize variability in microbial and biochemical properties (Jose et al., 2018). The samples were obtained under sterile conditions and transported at low temperatures (4°C) to ensure their preservation for subsequent analysis. After serial dilution, the sample was plated on MRS (de Man, Rogosa, and Sharpe agar media, HiMedia, India) agar plates and incubated at 37°C for 24 h at 5% CO_2_ incubator. Colonies exhibiting various morphologies were selected and subjected to morphological and biochemical assays (pH, 2–7.4; Temperature, 4°C–50°C; salt, 2–10%, phenol, 0.4%; 12 carbohydrates), including Gram-staining (HiMedia, India), catalase activity (Sigma-Aldrich, India), and cell morphology assessment (Cowan, 1948).

### 2.3 Molecular identification

Genomic DNA isolated using the phenol-chloroform isoamyl alcohol (PCI) method underwent PCR amplification with primers 27F and 1492R (Kumari V.B. et al., 2024). The protocol included initial denaturation at 95°C for 5 min, followed by 32 cycles of 95°C for 30 s (denaturation), 55°C for 30 s (annealing), and 72°C for 1 min (extension) 32 cycles of denaturation, annealing, and extension steps. PCR products were visualized on a 0.8% agarose gel stained with ethidium bromide. Sequences of the >1,500 bp 16S rRNA gene PCR products were analyzed using the BLAST program at NCBI, and accession numbers were obtained upon submission to GenBank (Kumari et al., 2023). Phylogenetic analysis was conducted using MEGA 11 software, employing the Maximum Likelihood method with the Tamura-Nei model for nucleotide substitutions and 1,000 bootstrap replicates for tree construction. Initial trees were generated using the BioNJ method based on pairwise distances, analyzing 6 nucleotide sequences comprising 1,570 positions with uniform rates among sites. The outgroup used was MT882202.1 *Lactiplantibacillus plantarum* strain DFRN2, a partial 16S rRNA gene sequence, which facilitated rooting the phylogenetic tree and clarifying evolutionary relationships among the taxa (Martiz et al., 2023).

### 2.4 Probiotic properties

As described by Zhong et al. (2021), acidic bile and simulated gastrointestinal juice tolerance were tested using MRS broth with ox gall salt (0.3 and 1%; pH 2; HiMedia, Mumbai). Simulated gastrointestinal tolerance was assessed using MRS broth with 3 g/L pepsin (pH 3, 2,500 U/mg) and 1 g/L trypsin (pH 8, 2,000 U/g) from HiMedia, Mumbai. To evaluate the survival of isolates in acidic bile salt, strains were plated on MRS agar and incubated at 37°C for 24 h. Surviving cell counts were recorded after exposure to 0.3 and 1% acidic bile salt at 0, 2 and 4 h (Douillard et al., 2018). Cell surface hydrophobicity, autoaggregation, and coaggregation assays were performed Mirtič et al., (2018) and Lipan et al., (2020) to evaluate adhesion capacity, with results expressed as percentages. For *in vitro* adhesion (Fonseca et al., 2021) was followed, isolates were tested on HT-29 cells, using crystal violet staining and subsequent microscopic observation. Strains with more than 80% viability were selected for further analysis (Kumari et al., 2023). For antibacterial activity (Sreepathi et al., 2023) method was followed, by using an agar well diffusion method. Where 100 μL of overnight-cultured LAB isolates were placed in wells of LB (Luria-Bertani) agar plates inoculated at 37°C for 24 h with pathogens as shown in Supplementary Table 1, and the zone diameters were measured in millimeters (Sreepathi et al., 2023). Dhanani and Bagchi (2013) the described method for antibiotic susceptibility was assessed using the disc diffusion method on MRS medium, with inhibition zones measured after overnight incubation at 37°C, The antibiotic disc used is shown in Supplementary Table 2. (Kumari V. B. et al., 2022) procedure was used to determine the hemolytic activity, which was tested by culturing Neera isolates on blood agar (5%; sheep blood), and observing zone formation around colonies to classify hemolysis types (Kumari V. B. et al., 2022).

### 2.5 Enzymes inhibition of α-glucosidase and α-amylase

The α-glucosidase inhibitory activity was evaluated by mixing 100 μL of Intact cell (IC), Cell-free extract (CE), and Cell-free supernatant (CFS) with 0.7 mL of potassium phosphate buffer, then adding 0.1 mL of α-glucosidase and 0.1 mL of p-nitrophenyl-D-glucopyranoside, followed by incubation and measurement of OD (Optical density) at 405 nm (Huligere et al., 2024). For α-amylase inhibition, 0.5 mL of IC, CE, and CFS were incubated with α-amylase and starch, stopped with 3,5-dinitrosalicylic acid, heated, diluted, and the absorbance was recorded at 540 nm (Thermo Multiskan FC Microplate Reader) (Kumari V. B. et al., 2022).

### 2.6 Cell culture, cell viability, and differentiation, Oil-Red O staining, and quantitative real-time PCR

3T3-L1 preadipocytes (Passage no. 12) were obtained from the National Centre for Cell Science (NCCS, Mumbai, India) and cultured in Dulbecco’s Modified Eagle’s Medium (DMEM; GIBCO, CA, USA) supplemented with 10% fetal bovine serum (FBS; GIBCO, CA, USA) and antibiotics. The cells were maintained at 37°C in a 5% CO_2_ environment. For the experiment, 3T3-L1 cells were seeded in a 96-well plate with 15,000 cells per well and incubated (24 h; 37°C, 5% CO_2_). Afterward, the cells were treated with different concentrations of the extract and further incubated for 72 h (Lee et al., 2015). After treatment, 20 μL of MTT (3-(4,5-dimethylthiazol-2-yl)-2,5-diphenyltetrazolium bromide) stock solution (5 mg/mL) was added to each well, and the plate was incubated for 4 h. Following incubation, the MTT solution was removed, and 100 μL of DMSO was added to each well (Park et al., 2011). The IC50 was measured using an online calculator tool by AAT Bioquest (Calculator | AAT Bioquest IC50, 2023). The plate was left in the dark for 15 min with gentle shaking to solubilize the formazan crystals. Absorbance was then measured at 540 nm using a microplate reader. For adipocyte differentiation was induced using an MDI (dexamethasone, 3-isobutyl-1-methylxanthine, and insulin) mixture (Sigma-Aldrich, India) for 2 days, followed by insulin-only medium and then DMEM (Park et al., 2011).

On days 5, 8, and 12, cells were cleaned three times with PBS and subsequently fixed in 10% formalin at room temperature for 1 h. Following this, the cells were rinsed with deionized water, treated with 60% isopropanol for 10 min, and stained with Oil-Red O at room temperature for 15 min. To assess the staining, the cells were washed twice with 60% isopropanol for 5 min each with gentle agitation. Finally, the Oil-Red O dye was removed with 100% isopropanol, and absorbance was measured at 492 nm (KiBeom and GunSu, 2015). On the same days, total RNA was isolated using the RiboEx reagent (GeneAll, Cat. No. 302-001), followed by cDNA synthesis from 0.001 mg of RNA obtained using the Primer Script RT Reagent kit (Takara, Cat. No. RR037A). Real-time PCR was conducted using a Rotor-Gene Q system and the QuantiFast SYBR Green PCR kit (QIAGEN, Cat. No. 204054; initial activation at 95°C for 5 min, followed by 40 cycles of 95°C for 10 s and 60°C for 30 s), normalizing relative mRNA levels to β-Actin using the delta–delta Ct method (36) (Kim et al., 2020). Primer sequences are detailed in Table 1.

**Table 1:** Genes and their accession number for quantitative real-time PCR.

Gene name	Accession no.	Forward primer	Reverse primer
PPAR-γ	NM_001127330	CAAGAATACCAAAGTGCGATCAA	GAGCTGGGTCTTTTCAGAATAATAAG
C/EBPα	NM_001287521	CCCTTGCTTTTTGCACCTCC	TGCCCCCATTCTCCATGAAC
FAS	NM_007988	CAAGTGTCCACCAACAAGC	GGAGCGCAGGATAGACTCAC
Adiponectin	NM_009605	CGATCTATCCGTCGGTGGTC	ATCCCTTTCCCTCCTCCTCC
Glut-4	NM_001359114	ATCCGGAACCTGGAGGGGCC	CGGCCAGGCCCAACAGATGG
ActB	NM_007988	TAAGAGGAGGATGGTCGCGT	CTCAGACCTGGGCCATTCAG

### 2.7 Glucose consumption assay using CFS of *Levilactobacillus brevis* RAMULAB51

3T3-L1 adipocytes were cultured in 6-well plates (e.g., Tarsons Products, India) and serum-starved in DMEM (HiMedia Laboratories) for 16 h. Following serum starvation, the cells were washed three times with PBS (pH 7.4, HiMedia Laboratories) and then incubated for 30 min in either DMEM alone or DMEM with 100 nM insulin (Sigma-Aldrich India), or DMEM with CFS from *Levilactobacillus brevis* RAMULAB51 at concentrations of 250 μg/mL, 500 μg/mL, or 1,000 μg/mL to assess glucose uptake. Additionally, some cells were incubated with a combination of insulin and CFS. After this incubation, 0.0005 M 2-deoxy-D-[2,6–^3^H] glucose (1.5 μCi/well; for radioactive isotopes, Sigma-Aldrich India) was introduced, and the cells were incubated for an additional 15 min. The cells, processed in triplicate, were then washed four times with PBS containing 0.3 mM phloretin (Sigma-Aldrich India) to inhibit glucose transporters. Following the washes, the cells were lysed in 1 mL of 1 N NaOH (HiMedia Laboratories) and subjected to scintillation counting to determine glucose uptake. Additionally, 3T3-L1 adipocytes were serum-starved for 16 h in DMEM before being treated with CFS (250, 500, and 1,000 μg/mL) or CFS combined with insulin for 15, 30, and 240 min, or with insulin (100 nM) alone for 30 min, followed by an assay for 2-deoxyglucose uptake (Shen et al., 2014; Etesami et al., 2020).

### 2.8 Statistical analysis

All experiments were performed in triplicate, and results were reported as mean ± standard deviation (*M* ± SD). To compare the isolates, a one-way analysis of variance (ANOVA) was used, followed by Duncan’s multiple range test (Duncans’s MRT), analyzed with SPSS Software (Version 21.0, Chicago, IL, USA). A *p*-value of 0.05 or lower was considered statistically significant. Graphs were generated using GraphPad Prism version 8.0 (GraphPad Software Inc., San Diego, CA, USA).

## 3 Results and discussion

### 3.1 Biochemical assay of LAB strains

On the MRS plates, approximately 30 distinct colonies were obtained. Among these, five isolates were identified through Gram-staining as Gram-positive and catalase-negative, characteristics consistent with LAB. Gram-staining is crucial for identifying LAB by revealing their Gram-positive nature and catalase-negative traits, distinguishing them from other bacteria (Guo et al., 2009). Confirmatory cell morphology assessments, showing typical rod-shaped LAB cells, reinforce their classification within this bacterial group (de Melo Pereira et al., 2018). Understanding LAB morphology is essential for their differentiation and utilization in food fermentation and probiotics (Huligere et al., 2023).

The biochemical assays conducted at different temperatures (4–50°C) demonstrated that all five isolates exhibited optimal growth at 37°C. This temperature preference is significant as it reflects the environmental conditions typically encountered in food fermentation processes and human gastrointestinal tracts (Uysal et al., 2009). The ability of these isolates to thrive under various temperature conditions highlights their potential adaptability and robustness, which are desirable traits for industrial and probiotic applications (Rao et al., 2022). The tolerance of all five isolates to salt concentrations up to 4% is noteworthy as it indicates their ability to survive and potentially ferment in environments with moderate salt levels (Paludan-Muller et al., 1999).

The isolates exhibited phenol and pH tolerances ranging from 6.25 ± 0.11 to 9.15 ± 0.15 and 5.42 ± 0.02 to 9.90 ± 0.56, respectively, after 24 h incubation. Among them, RAMULAB51 demonstrated notable tolerance to both pH and phenol (Table 2). *Lactobacillus* spp. Tolerance to phenol and pH is important in various applications, where survival in acidic environments and resistance to chemical stressors like phenol are critical for their functionality and efficacy (Huligere et al., 2022). This characteristic is advantageous for their utilization in fermenting salty foods or in conditions where salt is used as a preservative (Kumari et al., 2022). The carbohydrate fermentation assays revealed varying levels of activity in response to different sugars, as detailed in Table 3. The primary end products of carbohydrate fermentation by LAB include lactic acid, acetic acid, and other organic acids, depending on the substrates fermented. RAMULAB51 ferments all sugars except D-xylose and L-xylose, likely producing lactic acid as the main product and possibly acetic acid. RAMULAB28 has a similar profile but ferments one less sugar, likely yielding both lactic and acetic acids. RAMULAB26 and RAMULAB27 can ferment several sugars, suggesting the production of both acids. In contrast, RAMULAB25 shows a limited profile, likely producing lactic acid only where fermentation occurs. This variability in sugar metabolism underscores the metabolic diversity among the isolates and suggests potential differences in their fermentation capabilities (Dellias et al., 2018). LAB’s carbohydrate utilization profiles are crucial for predicting their fermentation pathways and the range of food products they can produce (Kingston et al., 2010).

**Table 2:** Phenol tolerance and the growth of Neera isolates at different pH levels.

Isolates	Tolerance to phenol (CFU/mL)^*^		Growth at varying pH levels (CFU/mL)^*^			
	0 h	24 h	2	4	6	7.4
RAMULAB25	6.14 ± 0.02^b^	7.08 ± 0.01^b^	6.78 ± 0.11^c^	7.14 ± 0.22^b^	8.44 ± 0.07^c^	9.12 ± 0.02^b^
RAMULAB26	7.56 ± 0.11^d^	7.90 ± 0.43^c^	6.13 ± 0.01^b^	7.54 ± 0.03^d^	7.89 ± 0.01^a^	8.65 ± 0.05^a^
RAMULAB27	5.78 ± 0.01^a^	6.25 ± 0.11^a^	5.42 ± 0.02^a^	6.83 ± 0.01^a^	8.78 ± 0.04^d^	9.89 ± 0.03^d^
RAMULAB28	6.81 ± 0.14^c^	7.15 ± 0.04^b^	6.41 ± 0.12^b^	7.19 ± 0.09^b^	8.21 ± 0.31^b^	9.71 ± 0.01^c^
RAMULAB51	8.81 ± 0.56^e^	9.15 ± 0.15^d^	7.12 ± 0.24^d^	7.96 ± 0.23^d^	8.16 ± 0.11^b^	9.90 ± 0.56^d^

* *M* ± SD is shown. Significant differences (p ≤ 0.05) within columns are denoted by different letters (a–e) per Duncan’s MRT.

**Table 3:** Carbohydrate fermentation activity of LAB isolates in response to different sugars.

	Tests	Carbohydrates fermentation*											
		1	2	3	4	5	6	7	8	9	10	11	12
Isolates	RAMULAB25	+	−	−	+	+	+	+	−	−	−	−	+
	RAMULAB26	+	+	+	+	+	+	−	+	−	−	−	+
	RAMULAB27	+	−	−	+	+	+	+	−	−	−	+	−
	RAMULAB28	+	+	+	+	+	+	+	+	−	−	−	+
	RAMULAB51	+	+	+	+	+	+	+	+	−	−	−	+

*(+) indicates active fermentation; (−) indicates no fermentation; 1: Glucose, 2: Galactose, 3: Lactose, 4: Maltose, 5: Sucrose, 6: Fructose, 7: Mannose, 8: Mannitol, 9: Arabinose, 10: Starch, 11: D-xylose, 12: L-xylose.

### 3.2 Molecular identification

The sequences obtained from Neera isolates varied in length, ranging from 1,249 to 1,582 base pairs after amplification. The sequences of the isolates were submitted to GenBank, obtaining the accession numbers RAMULAB25 (*Lacticaseibacillus casei*, GenBank: OK376494.1), RAMULAB26 (*Lacticaseibacillus paracasei*, GenBank: OK376500.1), RAMULAB27 (*Limosilactobacillus fermentum*, GenBank: OK376497.1), RAMULAB28 (*Lacticaseibacillus paracasei*, GenBank: OK376502.1), and RAMULAB51 (*Levilactobacillus brevis*, GenBank: ON171686.1). *Levilactobacillus brevis* strains similar to RAMULAB51 have also been documented by Phani Kumari et al. (2024) and identified as *Levilactobacillus brevis* MYSN105 (Somashekaraiah et al., 2019).

**Figure 1:**
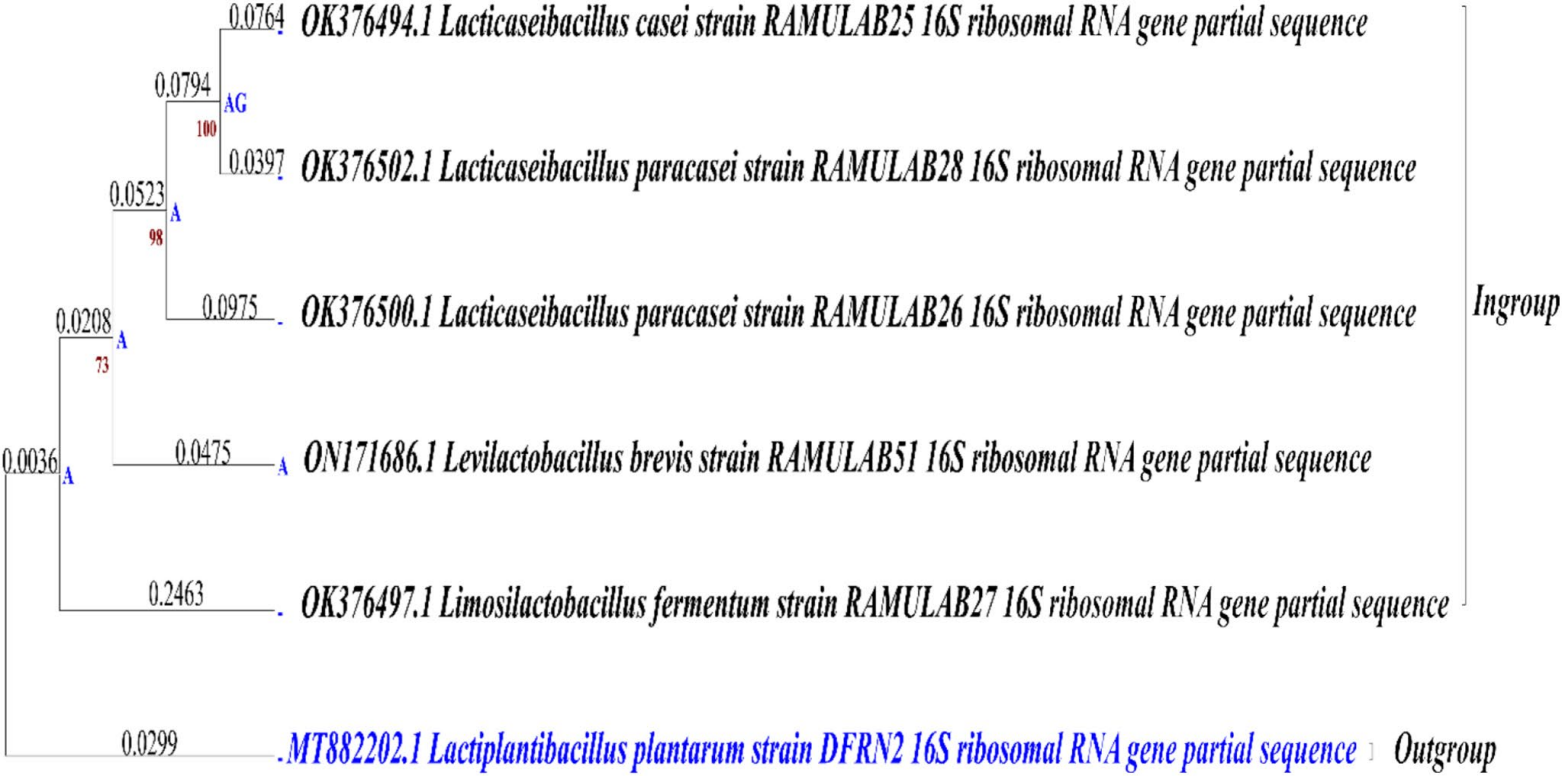
Comparison of phylogenetic trees for strains isolated from Neera (Ingroup) and reference outgroup, using maximum likelihood bootstrap 1,000 analysis of 16S rRNA sequences.

Using maximum likelihood with the Tamura-Nei model on nucleotide data from these eight taxa, covering 1,582 sites, phylogenetic analysis was performed. This comprehensive molecular characterization enhances our understanding of the evolutionary relationships and taxonomy of these bacteria, shedding light on their ecological roles in Neera and potentially other environmental contexts (Harris et al., 2017). The tree was constructed using the BioNJ method and was applied to a matrix of pairwise distances, estimated using the Tamura-Nei model, for the initial tree construction in the heuristic search (Figure 1). Ancestral nucleotide states were inferred and ranked by likelihood at each node, with only those states above a 5% probability threshold displayed. Examination of substitution rates highlighted key transitions, such as A->G and T->C, suggesting patterns of evolutionary change specific to the taxa under study. The phylogenetic analysis included an ingroup of five taxa, with **MT882202.1 *Lactiplantibacillus plantarum* strain DFRN2** designated as the outgroup. Using the outgroup sequence enabled the phylogenetic tree to be rooted, providing a reference point that clarified the evolutionary relationships among the ingroup taxa.

### 3.3 Probiotic properties

**Figure 2:**
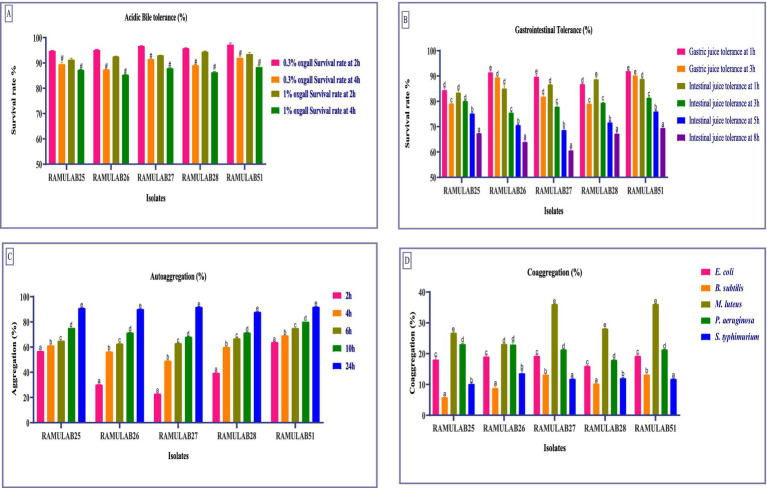
The survival rates of isolates at acidic pH 2 and various bile salt concentrations were assessed by incubating the strains for 2 and 4 h at 37°C with **(A)** 0.3 and 1% bile salt concentrations. Additionally, survival rates in **(B)** Gastric and intestinal juices were measured at 1, 3, 5, and 8 h, **(C)** Autoaggregation, and **(D)** Coaggregation of isolates with *M* ± SD compared using Duncan’s MRT and significant differences denoted by different superscripts (#, a–e) (*p* < 0.05).

In this study, we explored the flexibility of five isolates under conditions simulating the gastrointestinal environment. Cell membrane composition and efflux transporters help probiotics maintain cellular integrity and functionality in the presence of bile. Understanding these bile tolerance mechanisms is crucial as they highlight the role of probiotics in promoting gut health and aiding in the development of effective probiotic formulations (Ruiz et al., 2013). The survival rates of different Neera isolates were evaluated at two concentrations of oxgall (0.3 and 1%) and at two time points (2 h and 4 h). The highest survival rate was observed for the strain RAMULAB51 at 0.3% ox gall after 2 h, with a survival rate of 97.34% ± 0.54. On the other hand, the lowest survival rate was noted for the strain RAMULAB26 at 1% ox gall after 4 h, with a survival rate of 85.45% ± 0.15 (Figure 2A). Conversely, Phani kumari et al. study showed 10 isolates obtained from Neera samples from Choutuppal in Nalgonda, Telangana, exhibited a highly efficient 24-h survival rate of 1.8 when it was exposed to acid bile concentrations of 0.05 and 0.3%, respectively (Phani Kumari et al., 2024).

The tolerance tests for gastric and intestinal juices show notable differences among isolates. RAMULAB25 had high gastric juice tolerance at 1 h (84.53%) but decreased to 79.17% at 3 h. Its intestinal juice tolerance was 83.54% at 1 h, dropping to 67.48% at 8 h. RAMULAB26 exhibited the highest gastric juice tolerance, with 91.54% at 1 h and 89.52% at 3 h, and strong intestinal juice tolerance (85.13% at 1 h, 64.08% at 8 h). RAMULAB27 also showed good gastric juice tolerance (89.85% at 1 h, 81.98% at 3 h) and consistent intestinal juice tolerance, with 60.71% at 8 h. RAMULAB28 had 86.91% tolerance in gastric juice at 1 h and 88.78% in intestinal juice at 1 h, decreasing to 67.34% at 8 h. RAMULAB51 showed the highest overall tolerance, with 92.04% in gastric juice at 1 h and 88.89% in intestinal juice at 1 h, dropping to 69.58% at 8 h. Overall, RAMULAB51 had the highest tolerance across both juices, while RAMULAB26 and RAMULAB28 also demonstrated strong tolerance, especially in gastric juice (Figure 2B).

The cell surface hydrophobicity of the five bacterial isolates was measured and expressed as a percentage. The isolate RAMULAB51 exhibited the highest hydrophobicity at 78.14% ± 0.61, followed closely by RAMULAB25 with 76.21% ± 0.06. On the other hand, RAMULAB27 had the lowest hydrophobicity at 63.95% ± 0.04. The isolates RAMULAB28 and RAMULAB26 showed intermediate levels of hydrophobicity, with values of 72.05% ± 0.09 and 67.14% ± 0.12, respectively (Table 4). In Farid et al.’s study, the highest hydrophobicity observed among the LAB strains was 56.65%, with even the most adherent strain (WFA1) showing an adherence percentage of 55.48% (Farid et al., 2021). Reuben et al.’s findings also indicated lower hydrophobicity values, with isolates from crops showing a range of 70.0 ± 2.84–71.0 ± 8.48, while isolates from the intestine had values ranging from 40.5 ± 12.02 to 61.5 ± 3.54 (Reuben et al., 2019). In our study, significantly higher hydrophobicity levels, with RAMULAB51 reaching 78.14% ± 0.61, which is considerably above the values observed in both Farid et al.’s and Reuben et al.’s studies (Reuben et al., 2019; Farid et al., 2021). Even the isolate with the lowest hydrophobicity in your study, RAMULAB27, exhibited 63.95% ± 0.04, which is higher than the highest value reported by Farid et al. and comparable to the higher range of Rueben et al.’s isolates. Our results indicate a stronger hydrophobic interaction, suggesting potentially better adhesion capabilities of our bacterial isolates to surfaces, which could be advantageous in applications where high adhesion is desired.

**Table 4:** Assessment of cell surface hydrophobicity of Neera isolates.

Isolates	Hydrophobicity (%)^*^
RAMULAB25	76.21 ± 0.06^d^
RAMULAB26	67.14 ± 0.12^b^
RAMULAB27	63.95 ± 0.04^a^
RAMULAB28	72.05 ± 0.09^c^
RAMULAB51	78.14 ± 0.61^d^

* The mean values of the results are presented as *M* ± SD. To identify significant differences between the means, Duncan’s MRT was used. *M* ± SD within a column that is labeled with different alphabetical letters (a–d) denote statistically significant differences (*p* ≤ 0.05).

The auto-aggregation ability of five strains was assessed over a time course of 2, 4, 6, 10, and 24 h. The highest auto-aggregation was observed in strain RAMULAB51, which reached 92.23% ± 0.04 at 24 h. Conversely, the lowest auto-aggregation was recorded in strain RAMULAB27 at the 2-h mark, with a value of 23.13% ± 0.12. Across the strains, auto-aggregation generally increased with time. RAMULAB51 consistently showed higher aggregation across all time points, starting from 64.13% ± 0.04 at 2 h to the peak at 24 h (Figure 2C). In contrast, RAMULAB27 exhibited lower initial auto-aggregation but also showed a significant increase over time, ultimately reaching 92.01% ± 0.02 at 24 h. This suggests that although RAMULAB27 may require more time to establish aggregation, it can ultimately form strong communities, which could be beneficial in prolonged gastrointestinal colonization.

Coaggregation with other bacterial species is critical for inhibiting pathogen colonization through competitive exclusion and the formation of mixed-species biofilms (Vlková et al., 2008). The co-aggregation ability of the five RAMULAB isolates with various bacterial species was assessed and is reported as a percentage. RAMULAB27 and RAMULAB51 exhibited the highest co-aggregation percentages with *Bacillus subtilis*, achieving 13.23% ± 0.17. These two isolates also showed significant co-aggregation with *Micrococcus luteus*, both reaching 36.16% ± 0.05, This strong coaggregation indicates a high potential for RAMULAB27 and RAMULAB51 to interact with and possibly outcompete other microbial species in the gut, which is a desirable trait for probiotics. Among the other isolates, RAMULAB25 showed the highest co-aggregation with *Micrococcus luteus* at 26.82% ± 0.16, and with *Pseudomonas aeruginosa* at 23.14% ± 0.28. RAMULAB26 demonstrated the highest co-aggregation with *Salmonella typhimurium* at 13.69% ± 0.98 and with *Escherichia coli* at 19.11% ± 0.45. RAMULAB28 displayed moderate co-aggregation with *Micrococcus luteus* (28.18% ± 0.03) and lower co-aggregation percentages with the other tested bacterial species. The maximum co-aggregation was observed between RAMULAB27 and RAMULAB51 with *Micrococcus luteus*, while the lowest co-aggregation was noted between RAMULAB25 and *Bacillus subtilis* at 5.89% ± 0.03 (Figure 2D).

The auto-aggregation and co-aggregation abilities of LAB strains provide valuable insights into their potential as probiotic candidates (Hojjati et al., 2020). Comparing these results with previous studies reveals important similarities and differences in aggregation behaviors (Li et al., 2015). Our study findings align with the outcomes reported by Ramos et al., who observed the highest auto-aggregation in *L. plantarum* SAU96 and *L. fermentum* CH58 at 61.9 and 55.1%, respectively (Ramos et al., 2013). Our study’s strains show much higher auto-aggregation percentages, particularly RAMULAB51, which surpasses the highest values observed by Ramos et al. The gradual increase in auto-aggregation over time, as seen with RAMULAB27, also echoes the trend reported by Kumari et al., where prolonged incubation led to enhanced auto-aggregation (Kumari V B et al., 2024). This characteristic is beneficial for maintaining a strong presence in the gut environment. Co-aggregation with other bacterial species is essential for competitive exclusion and biofilm formation, which can inhibit pathogen colonization. Our results, in comparison, Li et al. reported that *L. sali*var*ius* M2-71 had the highest auto-aggregation of 95.6% at 24 h and demonstrated significant co-aggregation with enteropathogenic *E. coli* and *Salmonella typhimurium*. RAMULAB strains show high co-aggregation with *Micrococcus luteus* and varying levels with other species, reflecting a broader range of interactions compared to the more specific co-aggregation patterns observed by Li et al. (2021). The high auto-aggregation percentages observed in your study suggest that RAMULAB strains are strong candidates for probiotic use, with the potential for effective colonization and interaction with other microbial species in the gut. This enhances their ability to maintain a beneficial presence and potentially inhibit pathogen colonization (Kazou et al., 2018).

The adhesion percentages of five bacterial isolates to HT-29 cells, a human colorectal cancer cell line used to study bacterial interactions with intestinal epithelial cells, reveal varying adhesion abilities. **RAMULAB51** exhibits the highest adhesion at 79.45% ± 0.01%, indicating its superior ability to adhere to these cells. **RAMULAB28** follows with a high adhesion percentage of 76.02% ± 0.31%. **RAMULAB26** and **RAMULAB27** show moderate adhesion levels, at 71.96% ± 0.04 and 66.13% ± 0.02%, respectively. **RAMULAB25** has the lowest adhesion at 64.65% ± 0.01%. Table 5 findings suggest that RAMULAB51 and RAMULAB28 are most effective for applications requiring strong bacterial adhesion to intestinal cells, whereas RAMULAB25’s lower adhesion capability may limit its effectiveness in such contexts.

**Table 5:** Adhesion was assessed based on the percentage of Neera isolates that adhered to HT-29 cells.

Isolates	HT-29 adhesion (%)^*^
RAMULAB25	64.65 ± 0.01^a^
RAMULAB26	71.96 ± 0.04^c^
RAMULAB27	66.13 ± 0.02^b^
RAMULAB28	76.02 ± 0.31^d^
RAMULAB51	79.45 ± 0.01^d^

* The values of the results are presented as *M* ± SD. To identify significant differences between the means, Duncan’s MRT was used. This means that a column that is labeled with different alphabetical letters (a–d) designate statistically significant differences (*p* ≤ 0.05).

In comparison to other studies, such as those by Fonseca et al., where the adhesion of isolates did not vary significantly from that of the control strain *L. paracasei* LBC-81 on HT-29 cells, our findings reveal notably higher adhesion percentages (Fonseca et al., 2021). Fonseca et al. observed that while *L. paracasei* CCMA 0505 had higher adhesion to Caco-2 cells compared to the control strain, the differences on HT-29 cells were not statistically significant (Fonseca et al., 2021). This contrast highlights that RAMULAB51 and RAMULAB28 may have enhanced adhesion properties that are not only strain-specific but also cell-line-dependent.

In the study by Dhanani et al., *Lactobacilli* strains exhibited notable antagonistic effects on *E. coli* O26 adhesion to HT-29 cells. Specifically, *L. rhamnosus* GG and *L. plantarum* CS24.2 reduced *E. coli* adhesion through mechanisms such as competitive inhibition, adhesion interference, and displacement assays, indicating a competitive advantage in adhering to intestinal epithelial cells and potentially displacing pathogens (Dhanani and Bagchi, 2013). The strong adhesion abilities of RAMULAB51 and RAMULAB28 observed in our study suggest that these strains might also be effective in outcompeting pathogenic bacteria, similar to the effects seen *with L. rhamnosus* GG and *L. plantarum* CS24.2. These findings underscore the importance of strain-specific characteristics and cell-line interactions in evaluating bacterial adhesion and their potential applications in preventing or managing intestinal infections.

Table 6 data reveals the inhibition zones (in millimeters) for various bacterial isolates against different pathogens, highlighting the effectiveness of each isolate. **RAMULAB25** demonstrates moderate inhibition with the most substantial zone against *M. luteus* (20 mm) and no inhibition against *K. aerogenes*. **RAMULAB26** shows broad effectiveness, particularly against *P. aeruginosa* and *M. luteus* (21 mm each), but no activity against *K. aerogenes*. **RAMULAB27** exhibits varied inhibition patterns, with the highest zone against *P. aeruginosa* (20 mm) and the lowest against *K. pneumoniae* (5 mm). **RAMULAB28** displays significant inhibition against *M. luteus* (22 mm) and *E. coli* (13 mm), with no inhibition of *K. aerogenes*. **RAMULAB51** shows moderate inhibition across pathogens, with the largest zones against *M. luteus* (24 mm) and the smallest against *B. subtilis* (6 mm). The overall results suggest that different isolates have varying degrees of effectiveness, ranging from broad-spectrum inhibition to more specific activity. In comparing our results with those reported by Phani Kumari et al., it is evident that both studies reveal variability in antibacterial activity among different isolates (Phani Kumari et al., 2024). Our data shows that RAMULAB isolates exhibit a range of effectiveness, with RAMULAB26 and RAMULAB28 demonstrating significant inhibition against multiple pathogens, similar to the high activity observed for Isolate 3 against *Pseudomonas* in Phani Kumari et al.’s study. While our isolates generally showed a broader spectrum of activity, including notable inhibition against *M. luteus* and *E. coli,* Phani Kumari et al.’s isolates were more selective, with specific isolates exhibiting pronounced activity against *Pseudomonas* and *Klebsiella pneumonia*. Both studies indicate that some isolates show no activity against certain pathogens, highlighting the specificity and variability in antibacterial efficacy. This comparison underscores the diverse potential of bacterial isolates in antimicrobial applications and suggests that further exploration of their mechanisms and applications is warranted (Table 6).

**Table 6:** Neera isolates antibacterial activity against the pathogens.

Isolates	Pathogens								
	*K. pneumoniae*	*E. coli*	*B. cereus*	*B. subtilis*	*K. aerogenes*	*M. luteus*	*P. aeruginosa*	*P. florescens*	*S. aureus*
RAMULAB25	7	10	9	6	0	20	15	16	12
RAMULAB26	8	12	7	9	0	21	21	15	14
RAMULAB27	5	11	8	8	6	19	20	17	11
RAMULAB28	6	13	9	7	0	22	18	13	15
RAMULAB51	7	12	8	6	0	24	19	11	14

The values presented are zones of inhibition measured in millimeters. Interpretation of the results is as follows: 0 indicates no inhibition, less than 7 mm signifies weak activity, 9–15 mm denotes good activity, and greater than 15 mm represents strong activity.

The antibiotic susceptibility testing revealed that all bacterial isolates (**RAMULAB25**, **RAMULAB26**, **RAMULAB27**, **RAMULAB28**, and **RAMULAB51**) were sensitive to Ampicillin (AMP), Azithromycin (AZM), Streptomycin (STR), and Tetracycline (TET), with inhibitory zones exceeding the standard thresholds for sensitivity. Specifically, they showed high sensitivity to Ampicillin, Azithromycin, Streptomycin, and Tetracycline, with zones surpassing ≥17 mm, ≥13 mm, ≥15 mm, and ≥19 mm, respectively. In contrast, all isolates were resistant to Methicillin (MET) and Vancomycin (V), as indicated by inhibitory zones falling below or equal to the thresholds for resistance, i.e., ≤17 mm for Methicillin and ≤14 mm for Vancomycin. These findings highlight a clear pattern (Table 7). This is consistent with findings from Hana et al., who noted general sensitivity to certain antibiotics among *Lactobacillus* strains. However, our isolates were uniformly resistant to Methicillin (MET) and Vancomycin (V), reflecting a broader trend of resistance to these antibiotics. This mirrors previous observations where resistance to antibiotics like Methicillin and Vancomycin was common in *Lactobacillus* species, possibly due to the inherent resistance mechanisms and thicker cell walls of Gram-positive bacteria, as discussed by Hana et al. (2015). The distinct resistance patterns against these antibiotics further underscore the variability in antibiotic sensitivity among bacterial isolates, highlighting the importance of specific susceptibility testing for effective treatment and management.

**Table 7:** Antibiotic susceptibility testing of the Neera isolates was conducted to evaluate resistance and sensitivity, following the guidelines set by CLSI (2018).

Sl. No.	1	2	3	4	5	6
Antibiotic	Ampicillin (AMP)	Azithromycin (AZM)	Methicillin (MET)	Streptomycin (STR)	Tetracycline (TET)	Vancomycin (V)
The inhibitory zone (S/R mm)	(≥17/≤14)	(≥13/≤12)	(≥22/≤17)	(≥15/≤12)	(≥19/≤14)	(≥17/≤14)
RAMULAB25	Sens	Sens	Resi	Sens	Sens	Resi
RAMULAB26	Sens	Sens	Resi	Sens	Sens	Resi
RAMULAB27	Sens	Sens	Resi	Sens	Sens	Resi
RAMULAB28	Sens	Sens	Resi	Sens	Sens	Resi
RAMULAB51	Sens	Sens	Resi	Sens	Sens	Resi

Sens, Sensitive; Resi, Resistance.

The safety evaluation of the five Neera LAB isolates revealed that they were classified as γ-hemolytic after 48 h of incubation at 37°C, with no zones observed around the colonies. This finding indicates that the isolates are safe for use, making them suitable for probiotic applications. The γ-hemolytic classification means that these isolates do not cause hemolysis of red blood cells, which is a key indicator of reduced pathogenic risk. Such a classification is essential for verifying the safety of probiotics, as hemolytic activity can often signal potential virulence. This γ-hemolytic result is consistent with findings from previous studies (Somashekaraiah et al., 2019; Martiz et al., 2023; Kumari V.B. et al., 2024).

### 3.4 Enzymes inhibition of α-glucosidase and α-amylase

Diabetes, characterized by high blood glucose levels, requires effective strategies to manage postprandial glucose spikes (Musso et al., 2010). One promising approach involves inhibiting intestinal α-glucosidase, which can slow down carbohydrate digestion and absorption, thereby reducing postprandial hyperglycemia (Ramu et al., 2015). Recent research has focused on finding effective α-glucosidase inhibitors from diverse sources, including plant extracts and various foods (Kim et al., 2011; Patil et al., 2021).

**Figure 3:**
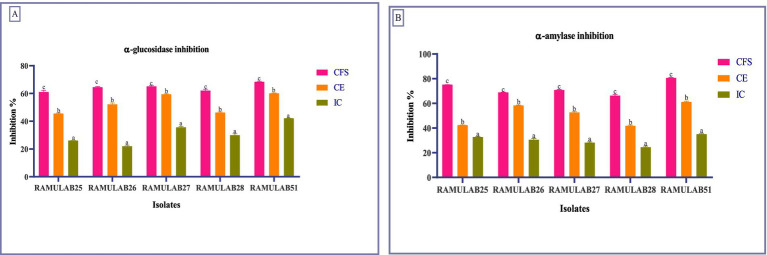
Enzymes inhibition by the isolates against α-glucosidase **(A)** and α-amylase **(B)**. Data are articulated as *M* ± SD. Duncan’s MRT indicates significant differences among means within the same column, with different letters (a–c) representing statistically distinct groups (*p* ≤ 0.05).

In our study, RAMULAB51 emerged as the most potent inhibitor among the tested isolates, achieving α-glucosidase inhibition rates of 68.45% for CS, 60.18% for CE, and 42.15% for IC. On the other hand, RAMULAB25 showed lower inhibition levels, with 61.12% for CS, 45.56% for CE, and 26.13% for IC (Figure 3A). These findings surpass those from earlier studies, where LAB strains like *L. rhamnosus* GG and *B. bifidum* F-35 showed maximum inhibitory activities of 29.57 and 21.82%, respectively (Chen et al., 2014). Our results align with the notion that inhibiting α-glucosidase is an effective method to mitigate carbohydrate absorption and control hyperglycemia (Ron et al., 2002; Oboh et al., 2016). Also, two strains, MBEL1361 and MBEL1397, demonstrated α-glucosidase inhibitory effects sourced from kimchi, exhibited an inhibition of 3.91 ± 0.25%, which is about 2.3 times greater than the control, acarbose (Kwun et al., 2020). Chen et al. (2014) also documented α-glucosidase inhibition in strains like *L. casei, L. rhamnosus GG, L. bulgaricus, and L. plantarum,* with *L. casei* showing 0.14 ± 0.08% inhibition and *L. rhamnosus* GG demonstrating 3.63 ± 0.17% inhibition (Chen et al., 2014). Previous studies have reported varying degrees of α-glucosidase inhibition among LAB strains, with Zeng et al. (2016) noting inhibition levels ranging from 2.5 to 13.7% for strains such as *L. rhamnosus* and *L. plantarum* (Zeng et al., 2016).

For α-amylase inhibition, **RAMULAB51** again displayed the highest activity, with 80.74% for CS, 61.23% for CE, and 35.12% for IC. This is significantly higher than the previous reports where the highest α-amylase inhibition was 75.19% for *L. rhamnosus* GG (Chen et al., 2014). On the other hand, **RAMULAB28** exhibited the lowest inhibition, with 66.38% for CS, 41.84% for CE, and 24.54% for IC (Figure 3B). This contrasts with previous studies that showed lower inhibition rates, highlighting the efficacy of our isolates and their potential benefits in diabetes management (Talamond et al., 2002; Tirwa et al., 2020; Maradesha et al., 2022). Since the CFS of *Levilactobacillus brevis* RAMULAB51 demonstrated significant inhibitory activity against α-glucosidase and α-amylase enzymes, further assays were conducted to investigate its potential therapeutic effects. These subsequent experiments were designed to explore the efficacy of RAMULAB51 CFS in modulating enzyme activity, which may have implications for its use in managing conditions such as diabetes by influencing carbohydrate digestion and absorption.

### 3.5 Cell viability, and differentiation

**Figure 4:**
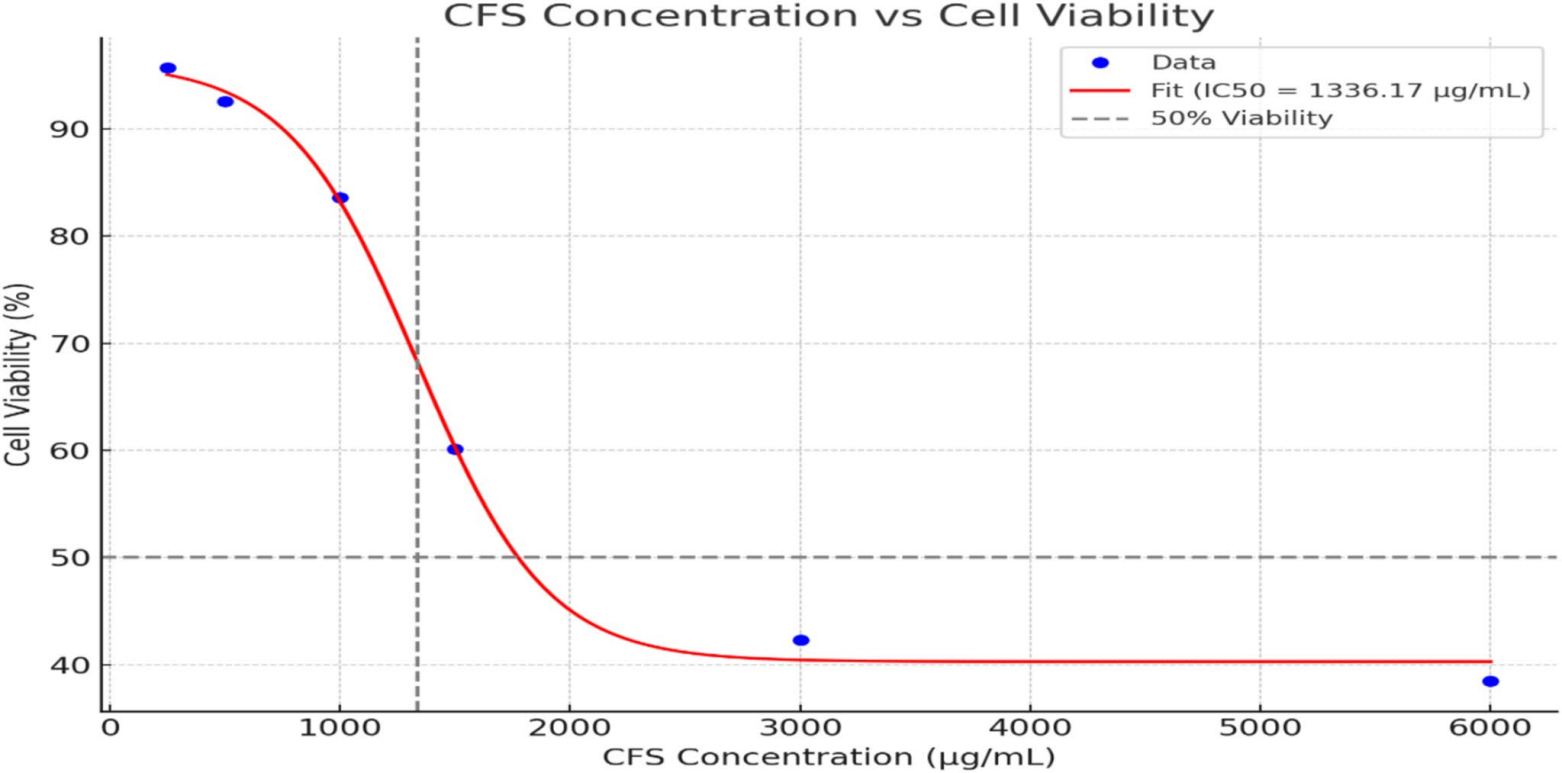
The effect of CFS from *Levilactobacillus brevis* RAMULAB51 on cell viability at various concentrations. The data points represent *M* ± SD of cell viability percentages. The sigmoidal curve fit shows a dose-dependent reduction in cell viability was observed with increasing concentrations of CFS. The IC50 value, where cell viability is reduced to 50%, is approximately 1,336.17 μg/mL, indicated by the vertical dashed line on the graph.

The effect of CFS from *Levilactobacillus brevis* RAMULAB51 on cell viability was assessed at various concentrations, as presented in Figure 4. At a concentration of 250 μg/mL, cell viability remained high at 95.7% ± 1.11, indicating minimal impact on cell health. As the concentration increased to 500 μg/mL, cell viability slightly decreased to 92.6% ± 1.52. At 1,000 μg/mL, a more pronounced reduction in cell viability was observed, with values dropping to 83.6% ± 0.11. Further increases in CFS concentration led to progressively lower cell viability, reaching 60.13% ± 0.01 at 1,500 μg/mL, 42.3% ± 0.05 at 3,000 μg/mL, and 38.5% ± 1.25 at 6,000 μg/mL. These results indicate a dose-dependent decrease in cell viability with higher concentrations of CFS from *L. brevis* RAMULAB51, highlighting the potential cytotoxic effects of the supernatant at elevated concentrations. The graph illustrates the relationship between CFS concentration and cell viability, with a sigmoidal curve fitting the data. The IC50 value (Figure 4), representing the concentration at which cell viability is reduced to 50%, is approximately 1,336.17 μg/mL, as indicated by the vertical dashed line on the graph. This sigmoidal fit highlights the gradual decrease in cell viability with increasing CFS concentration and provides a quantitative measure of the concentration required to achieve a half-maximal inhibitory effect.

Table 8 presents the quantification of triglyceride (TG) and lipid accumulation via Oil-Red O staining at various concentrations of CFS from *Levilactobacillus brevis* RAMULAB51. On day 5, the absorbance values for the control group were 0.75 ± 0.05, which decreased progressively with increasing CFS concentrations. Specifically, CFS at 250 μg/mL resulted in an absorbance of 0.68 ± 0.04, while CFS at 500 μg/mL and 1,000 μg/mL yielded lower absorbances of 0.55 ± 0.03 and 0.40 ± 0.02, respectively. This trend continued on days 8 and 12, with absorbance values decreasing further in a dose-dependent manner. Relative lipid content, compared to the control group, was significantly reduced in treated groups, with 86% for CFS 250 μg/mL, 68% for CFS 500 μg/mL, and 52% for CFS 1,000 μg/mL. Cytoplasmic TG content also decreased with higher concentrations of CFS, from 50.5 ± 2.3 μg/mg protein in the control group to 28.2 ± 1.5 μg/mg protein in the 1,000 μg/mL CFS-treated group.

**Table 8:** Quantification of triglyceride and lipid accumulation via Oil-Red O staining.

Treatment	Concentration (μg/mL)	Day 5 absorbance (OD)^*^	Day 8 absorbance (OD)^*^	Day 12 absorbance (OD)^*^	Relative lipid content (%)^*^	Cytoplasmic TG content (μg/mg protein)^*^
Control	–	0.75 ± 0.15 ^d^	0.85 ± 0.16^d^	0.92 ± 0.07^d^	100 ± 0.01^d^	50.5 ± 2.3^d^
CFS 250 μg/mL	250	0.68 ± 0.04^c^	0.75 ± 0.01^c^	0.80 ± 0.03^c^	86 ± 0.04^c^	42.0 ± 1.9^c^
CFS 500 μg/mL	500	0.55 ± 0.23^b^	0.60 ± 0.42^b^	0.65 ± 0.01^b^	68 ± 0.04^b^	35.8 ± 1.8^b^
CFS 1,000 μg/mL	1,000	0.40 ± 0.02^a^	0.45 ± 0.03^a^	0.50 ± 0.04^a^	52 ± 0.02^a^	28.2 ± 1.5^a^

* Absorbance values were measured at 520 nm. Relative lipid content is defined as a percentage of the control group’s values, and cytoplasmic triglyceride (TG) content is normalized to total protein concentration. Values are shown as the *M* ± SD. Duncan’s MRT was employed to identify significant differences among the means in the same column. Different letters (a–d) indicate statistically distinct groups (*p* ≤ 0.05).

**Figure 5:**
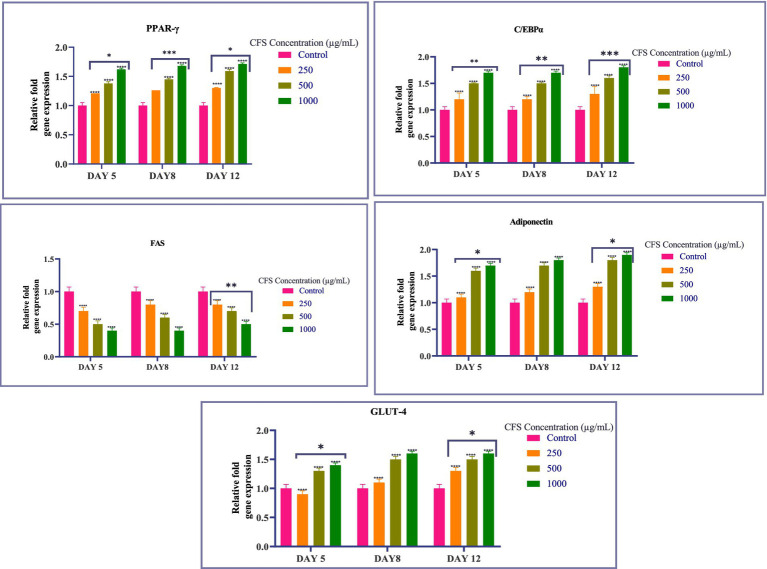
Graph showing the relative expression levels of PPAR-γ, C/EBPα, FAS, Adiponectin, and Glut-4 in 3T3-L1 adipocytes treated with varying concentrations of CFS on days 5, 8, and 12. Values existing, as the *M* ± SD, normalized to β-actin levels. Duncan’s MRT was employed to identify significant differences. Statistical significance is denoted as follows: ****p < 0.0001, ***p < 0.001, **p < 0.01, *p < 0.05.

To assess the impact of CFS on adipogenesis, we analyzed the expression of crucial adipogenic transcription factors like PPAR-γ and C/EBPα, as well as adipocyte-specific genes such as FAS, Adiponectin, and Glut-4. Quantitative real-time PCR was used to measure gene expression levels, and the results are presented in the following graph (Figure 5). Our findings revealed a dose-dependent **upregulation** of most adipogenic markers as the concentration of CFS increased. Specifically, the expression of PPAR-γ, C/EBPα, Adiponectin, and Glut-4 showed a consistent increase with higher doses of CFS. This upregulation was observed across multiple time points (Day 5, Day 8, and Day 12), with a more pronounced effect at the higher concentrations of 500 μg/mL and 1,000 μg/mL. PPAR-γ serves as a key regulator of adipogenesis, significantly influencing the process of differentiating preadipocytes into mature adipocytes. The observed upregulation of PPAR-γ in a dose-dependent manner indicates that CFS promotes adipocyte differentiation. This suggests that *Levilactobacillus brevis* RAMULAB51 may enhance adipogenesis through the activation of PPAR-γ, which is critical for lipid metabolism and the maintenance of insulin sensitivity in adipose tissue (Ortuño Sahagún et al., 2012). C/EBPα is another important transcription factor involved in adipocyte differentiation, working synergistically with PPAR-γ. The upregulation of C/EBPα alongside PPAR-γ further supports the hypothesis that CFS facilitates adipogenesis by promoting the expression of key transcription factors that drive the conversion of preadipocytes to adipocytes (Rai et al., 2021). This coordination between PPAR-γ and C/EBPα is essential for the activation of adipocyte-specific genes (Madsen et al., 2014). Adiponectin is a hormone secreted by adipocytes that plays a role in regulating glucose levels and fatty acid breakdown (Oh et al., 2021). The upregulation of Adiponectin with increasing CFS concentrations indicates that CFS not only promotes adipogenesis but may also enhance the metabolic functionality of adipocytes (Oh et al., 2021) Higher levels of Adiponectin are linked to enhanced insulin sensitivity and possess anti-inflammatory effects, which could have positive implications for metabolic health (Zulkawi et al., 2018). Glut-4 is responsible for insulin-stimulated glucose uptake in adipocytes and muscle cells. The upregulation of Glut-4 suggests that CFS may improve glucose metabolism in adipocytes by enhancing insulin sensitivity. This could be particularly significant in the context of metabolic disorders such as type 2 diabetes, where impaired glucose uptake is a key issue (Kord et al., 2020). FAS is a key enzyme intricate in lipogenesis, responsible for the synthesis of fatty acids. The downregulation of FAS in response to CFS treatment suggests that while CFS promotes adipogenesis and the expression of other adipogenic markers, it may inhibit fatty acid synthesis (Chandra et al., 2008). This downregulation could indicate a protective mechanism against excessive lipid accumulation, potentially preventing adipocyte hypertrophy and reducing the risk of obesity-related complications (Rai et al., 2021). The differential regulation of these genes by CFS from *Levilactobacillus brevis* RAMULAB51 highlights its complex role in modulating adipogenesis. While CFS promotes the expression of key transcription factors and genes involved in adipocyte differentiation and metabolism (PPAR-γ, Adiponectin, C/EBPα, and Glut-4), it simultaneously downregulates FAS, suggesting a balanced approach to lipid accumulation (Majithia et al., 2014; Mu et al., 2020). This regulatory effect may have therapeutic potential in managing metabolic diseases by enhancing adipocyte function while preventing excessive fat accumulation. The results suggest that CFS from *Levilactobacillus brevis* RAMULAB51 effectively inhibits adipogenesis by reducing lipid accumulation and downregulating the expression of key adipogenic transcription factors and adipocyte-specific genes (Figure 5). The promising findings regarding RAMULAB51’s enzyme-inhibiting capabilities and its probiotic properties suggest its potential role as an adjunct therapy in diabetes management. By effectively modulating postprandial hyperglycemia, RAMULAB51 could complement traditional diabetes medications, enhancing glycemic control and supporting overall metabolic health. Furthermore, its application as a preventive therapy may provide an innovative approach to reducing the risk of diabetes onset in at-risk populations. Future research should focus on clinical trials to validate these benefits, ultimately paving the way for RAMULAB51’s integration into holistic diabetes care strategies.

### 3.6 Glucose uptake assay

**Figure 6:**
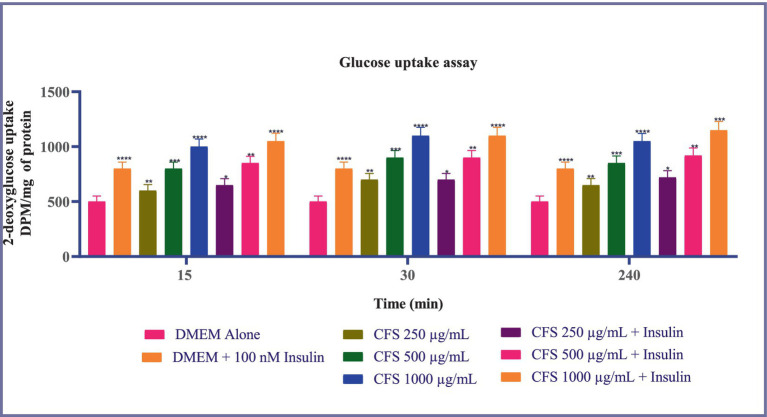
Glucose uptake in 3T3-L1 adipocytes subjected to various concentrations of CFS (500 μg/mL and 1,000 μg/mL) and insulin (100 nM) across different time points (15, 30, and 240 min). Data existing, as the *M* ± SD, normalized to β-actin levels. Duncan’s MRT was employed to identify significant differences. Statistical significance is denoted as follows: ****p < 0.0001, ***p < 0.001, **p < 0.01, *p < 0.05.

Glucose uptake across all time points-15, 30, and 240 min-the common observation is that CFS 1,000 μg/mL consistently results in the highest glucose uptake compared to other treatments. This effect is significant and maintains a higher level of glucose uptake throughout the experiment. Insulin (100 nM) consistently enhances glucose uptake in a manner comparable to the highest CFS concentrations. CFS 500 μg/mL also shows a significant increase in glucose uptake relative to DMEM alone at all time points, although it is less pronounced than CFS 1,000 μg/mL. Furthermore, the concentration-dependent increase in glucose uptake by CFS and the effectiveness of insulin as a positive control are observed uniformly across the different time points (Figure 6). These findings indicate that CFS significantly enhances glucose uptake in a concentration-dependent manner, with the most pronounced effect observed at 30 min and sustained at 240 min. The consistent performance of CFS 1,000 μg/mL and the reliability of insulin as a positive control reinforce the potential of CFS as a valuable agent for improving glucose metabolism, which could have implications for therapeutic strategies in managing glucose-related disorders. Additional research is required to clarify the underlying mechanisms and to investigate, the potential clinical applications of CFS in glucose regulation.

## 4 Summary and conclusion

This study highlights the significant potential of the CFS from *Levilactobacillus brevis* RAMULAB51 in managing glucose-related disorders. The CFS demonstrated remarkable inhibitory activity against α-glucosidase and α-amylase enzymes, which are crucial for carbohydrate digestion. This inhibition can help control postprandial glucose spikes, offering a promising alternative to conventional treatments. The study also revealed that the CFS affects cell viability in a concentration-dependent manner, with higher concentrations potentially causing cytotoxic effects. This suggests that while RAMULAB51’s CFS can be beneficial, care must be taken with dosing to avoid adverse effects. Besides, RAMULAB51’s CFS was shown to effectively reduce lipid accumulation and modulate adipogenesis. It upregulated key adipogenic markers such as PPAR-γ, C/EBPα, Adiponectin, and Glut-4, while downregulating FAS, indicating a balanced approach to lipid metabolism. This modulation supports improved lipid metabolism and could help in managing conditions related to fat storage and metabolism. In addition, the CFS enhanced glucose uptake in 3T3-L1 adipocytes, with the most significant effects observed at 1,000 μg/mL. This suggests that RAMULAB51’s CFS has the potential to improve glucose metabolism, which is critical for diabetes management.

In conclusion, the findings underscore the therapeutic potential of RAMULAB51’s CFS by inhibiting α-glucosidase and α-amylase, RAMULAB51’s CFS reduces glucose absorption, which helps control blood sugar levels. This control positively impacts metabolic pathways, leading to the upregulation of adipogenic markers that support balanced fat metabolism and improved glucose uptake in adipocytes. Together, these effects suggest a comprehensive approach to managing glucose metabolism and lipid accumulation, contributing to overall metabolic health.

## Data Availability

The datasets presented in this study can be found in online repositories. The names of the repository/repositories and accession number(s) can be found in the article/Supplementary material.
