# Supplementary material for: Cell-free supernatant of Levilactobacillus brevis (RAMULAB51) from coconut inflorescence sap (Neera) enhances glucose uptake and PPAR-γ in 3T3-L1 adipocytes and inhibits α-glucosidase and α-amylase
Source: Front Microbiol. 2024 Dec 23;15:1497023. doi: 10.3389/fmicb.2024.1497023 (PMC11701883; doi:10.3389/fmicb.2024.1497023)
Supplement: Supplementary file 1 [file Table_1.docx]

**Supplementary Table 1:** List of pathogens used for antibacterial assay.

| Sl. No. | Pathogens | Accession Numbers |
| --- | --- | --- |
| 1 | *Bacillus cereus* | MTCC-1272 |
| 2 | *Bacillus subtilis* | MTCC1-0403 |
| 3 | *Escherichia coli* | MTCC-443 |
| 4 | *Klebsiella aerogenes* | MTCC-2822 |
| 5 | *Klebsiella pneumonia* | MTCC-10309 |
| 6 | *Micrococcus luteus* | MTCC-1809 |
| 7 | *Pseudomonas aeruginosa* | MTCC-424 |
| 8 | *Pseudomonas fluorescens* | MTCC-667 |
| 9 | *Salmonella typhimurium* | MTCC-98 |
| 10 | *Staphylococcus aureus* | MTCC-1144 |

**Supplementary Table 2:** List of antibiotic discs used for antibiotic assay.

| Sl. No. | µg / Discs * | Antibiotics |
| --- | --- | --- |
| 1 | 100 | Streptomycin |
| 2 | 30 | Vancomycin, Tetracycline |
| 3 | 15 | Azithromycin |
| 4 | 10 | Methicillin and Ampicillin |

*****Disc concentrations used based on following the guidelines set by CLSI, 2018.
